# Granular friction: Triggering large events with small vibrations

**DOI:** 10.1038/srep13455

**Published:** 2015-09-03

**Authors:** Henri Lastakowski, Jean-Christophe Géminard, Valérie Vidal

**Affiliations:** 1 Laboratoire de Physique, École Normale Supérieure de Lyon - CNRS, Université de Lyon, 46, Allée d’Italie, 69364 Lyon cedex 07, France

## Abstract

Triggering large-scale motion by imposing vibrations to a system can be encountered in many situations, from daily-life shaking of saltcellar to silo unclogging or dynamic earthquakes triggering. In the well-known situation of solid or granular friction, the acceleration of imposed vibrations has often been proposed as the governing parameter for the transition between stick-slip motion and continuous sliding. The threshold acceleration for the onset of continuous slip motion or system unjamming is usually found of the order of the gravitational acceleration. These conclusions are mostly drawn from numerical studies. Here, we investigate, in the laboratory, granular friction by shearing a layer of grains subjected to horizontal vibrations. We show that, in contrast with previous results, the quantity that controls the frictional properties is the characteristic velocity, and not the acceleration, of the imposed mechanical vibrations. Thus, when the system is statically loaded, the typical acceleration of the vibrations which trigger large slip events is much smaller than the gravitational acceleration. These results may be relevant to understand dynamic earthquake triggering by small ground perturbations.

Granular assemblies are athermal systems, often used as paradigms to study the dynamics of industrial or natural processes, such as fault gouge and earthquake nucleation^1^,^2^,^3^,^4^,^5^,^6^. When submitted to mechanical vibrations, granular media can behave as thermalized fluids when the vibrations are large enough, or remain in a solid-like state for small enough vibrations. Such a transition between jammed and unjammed states has been reported to occur when the peak-acceleration characterizing the vibration is of the order of the gravitational acceleration^7^,^8^.

The study of sheared granular layers is a classical way to probe the frictional properties of granular matter. At low shear velocity, a periodic stick-slip motion of the slider is observed, like in solid friction. When the pulling velocity is increased, the stick-slip motion becomes irregular, until the system reaches a continuous sliding regime^9^,^10^. Numerical simulations have shown that this transition can be achieved by imposing mechanical vibrations: the system, initially in the stick-slip regime, undergoes a continuous sliding above a given vibration threshold. The mechanical vibrations both reduce the friction when their amplitude increases^11^, and trigger an order/disorder transition in the dense granular assembly^7^,^12^,^13^.

However, many discrepancies remain on the pertinent quantity governing the transition between stick-slip motion and continuous sliding. Most studies point out the peak-acceleration of the imposed vibration as the governing parameter, with a transition threshold of the order of the gravitational acceleration^7^,^12^,^14^,^15^. These results are in contradiction with the very low acceleration value needed to unjam a granular hopper with vibrations^16^ or with the strain wave amplitude threshold, of the order of 10^−7^ to 10^−6^ for a moderate sized earthquake, often proposed for dynamic earthquake triggering^3^,^17^,^18^. The role of frequency, when considered, is still debated, whether in the dry^11^,^13^,^19^ or wet case^20^,^21^.

Here we report on the effects of horizontal harmonic-vibrations on a sheared granular medium, and systematically investigate the role played by the amplitude and frequency on the system dynamics. The results evidence that the velocity is the quantity adequate to account for the effects of vibrations on the frictional properties of the material.

## Experimental setup

**Figure 1:**
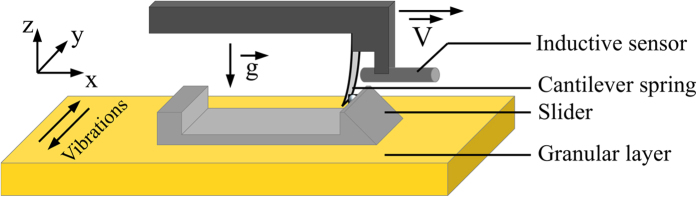
Sketch of the experimental setup. The slider is pulled at constant velocity, via a flexible blade, accross the upper surface of a granular layer. A shaker (not represented here) imposes horizontal vibrations of the whole in the transverse direction (along the *y*–axis).

To study the granular friction under vibration at the laboratory scale (Fig. 1), a slider is laid over a layer of spherical beads [sodosilicate glass, Wheelabrator, diameter *d* = (250 ± 23) *μ*m and density *ρ* = 2.31 × 10^3^ kg m^−3^, except when specified]. To ensure a good frictional contact between the slider and the material, one monolayer of grains, identical to those in the tank, is glued on the bottom surface. The slider mass is *m* = 30.7 g, and the area of the contact with the grains is 9 × 6 cm^2^. The slider is put in motion by means of a cantilever-spring, an aluminium blade (5 × 1 cm^2^) whose stiffness *k* can be varied by changing its thickness (from 0.25 to 0.4 mm). Almost vertical, its lower end is in ponctual contact with the slider whereas its upper end is embedded in a holding frame displaced horizontally at constant velocity *V*. A DC motor coupled with a reduction gear (5 N.m, 17 W, Crouzet) drives the linear-translation stage (Schnaefler Technologies Sechnr). A bellows seal transmits the torque from the motor to the stage, while avoiding mechanical vibrations. The velocity *V* ranges from 15 to 75 *μ*m s^−1^. An inductive sensor (IPRM 12I9505/S14, Baumer) measures the deflection of the blade and, thus, assesses the instantaneous force *F* applied to the slider. In the following, we denote 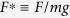 the dimensionless driving force, with *g* the gravitational acceleration.

**Figure 2:**
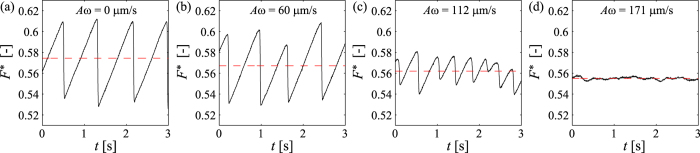
Dimensionless force F^*^ vs. time *t* for different amplitudes *A* of the imposed vibrations [*k* = 870 N/m, *f* = *ω*/2*π* = 280 Hz, *V* = 35 *μ*m s^−1^].

Reproducible experiments are ensured with the following protocol. We work at constant air humidity, *R*_H_ ≃ (37 ± 2)% and temperature, *T* ≃ 21.5 °C. The granular layer (50 × 15 cm^2^) is stirred with a brush, then levelled (thickness *h* ≃ 1 cm) with a squeegee which is moved at constant height along the sliding direction. We then impose a translation velocity *V* to the holding frame. In absence of external vibrations, in the range of pulling velocity *V* used in our experiments, the slider experiences a classical stick-slip motion characterized by a sawtooth variation of the force F^*^ in the stationary regime (Fig. 2a)^10^: In the stick phase, the slider does not move and the force F^*^ increases until 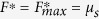, the static friction coefficient; Then, the slider starts moving forwards and F^*^ suddenly decreases. In average over this slip phase, 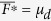, the dynamic friction coefficient.

Once the stick-slip regime is well-established, a mini-shaker (Brüel & Kjær, Type 4810 + amplifier 2706) clamped on the experimental tank imposes sinusoidal vibrations to the whole granular layer, in the horizontal plane, along a direction transverse to the slider motion. Three accelerometers (Dytran Instruments, Model #3035BG) embedded in the granular layer measure the three components of the local acceleration. We checked that the acceleration is sinusoidal, spatially homogeneous and anisotropic. Its main component is oriented in the transversal direction, as imposed by the shaker. We denote by *ω* the angular frequency and by *A* the amplitude of vibration in the transverse direction. In accordance, we define the acceleration 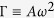, which remains smaller than *g* in our experimental conditions. Note that, in our experimental conditions, we do not observe any significant motion of the grains at the free surface.

## Transition to continuous sliding

**Figure 3:**
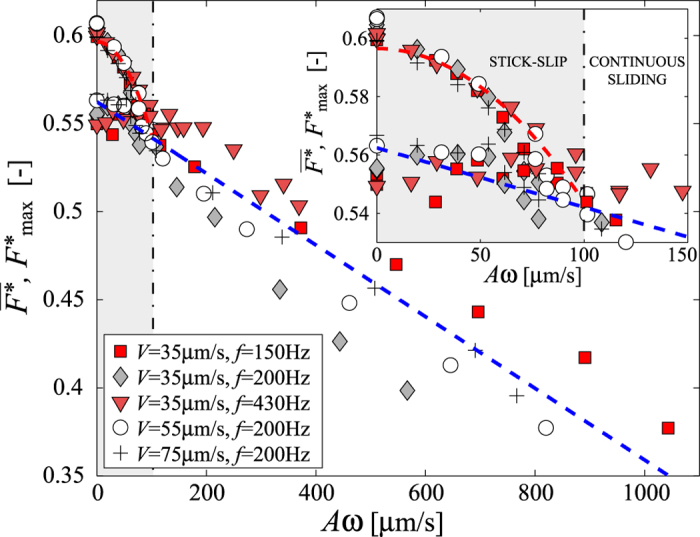
Maximum (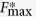) and average (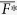) dimensionless force as a function of the vibration velocity (*Aω*) for different pulling velocity *V* and vibration frequency *f* [*k* = 870 N m^−1^]. The red and blue lines correspond to quadratic and linear fit of the data, respectively (see text). *Inset:* zoom on the same data showing the transition between stick-slip (light gray zone) and continuous sliding (white zone).

Increasing the amplitude of the vibrations, we observe an almost instantaneous decrease of both the amplitude of the stick-slip and the average friction force (Fig. 2). In other words, both 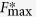 and 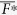 decrease when the intensity of vibration is increased (Fig. 3). Moreover, at a given *ω*, there exists a threshold in the vibration amplitude above which the stick-slip disappears, and the motion is continuous (Fig. 2d). Interestingly, all data gather on the same curve when displayed as a function of the velocity, *Aω* (Fig. 3, inset). This striking observation is the main result of our study.

The stick-slip amplitude, 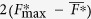, vanishes for the critical velocity *v*_*c*_ = (*Aω*)_*c*_ ≃ (100 ± 20) *μ*m s^−1^ for all *ω*. We first checked that the result does not depend on the depth of the granular bed (from 2 mm to 2 cm), in agreement with the experimental fact that the shear is localized in a thin region, a few particle diameters in thickness, below the slider^22^,^23^. Note also that, in our experimental conditions, the velocity of the slider is always much smaller than the critical velocity V^*^ associated with the transition to continuous sliding in absence of vibration^10^. The threshold vibration velocity, *v*_*c*_, does not significantly depend neither on the cantilever stiffness (*k* between 210 and 870 N m^−1^), vibration frequency, *f*, nor pulling velocity *V*, in the experimental range (Fig. 3). More surprisingly, it does not significantly depend neither on the diameter, polydispersity, density, nor shape of the grains. Indeed, experiments with spherical glass beads, 45–90 *μ*m in diameter, or ceramic grains, 425–600 *μ*m in typical size, display the same behaviour, with the same threshold velocity *v*_*c*_ to within 20%. We point out that the critical velocity *v*_*c*_ is thus associated with an acceleration Γ which is usually much smaller than *g*. For instance, for *f* = *ω*/(2*π*) = 200 Hz, Γ is of about 1% of *g*, thus much smaller than what is classicaly found, theoretically, for vertical vibrations of model frictional systems (Γ ≃ *g*)^15^ or, experimentally, for vertical or horizontal vibrations of granular systems (Γ ≃ 0.1 − 1*g*)^7^,^8^,^11^,^19^. Such small value reveals that, above the transition threshold, the grains are not in a fluidized state, although we observe a continuous slip of the slider.

**Figure 4:**
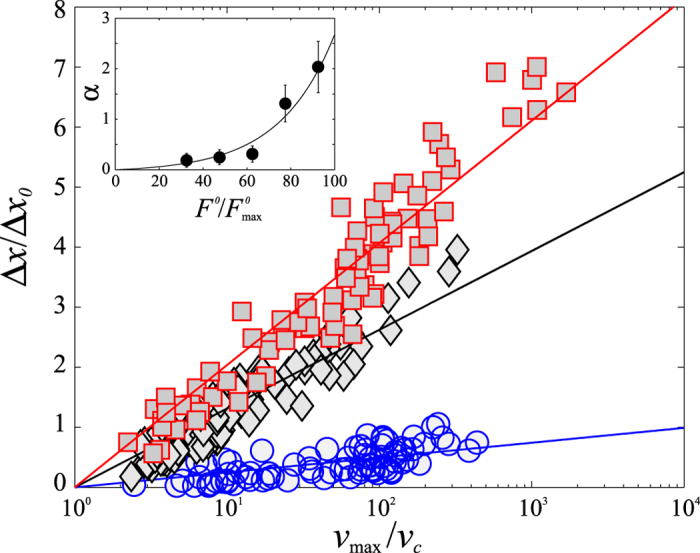
Response of the system to a single mechanical disturbance. Normalized slip distance (Δ*x*/Δ*x*_0_) as a function of the normalized maximum wave velocity *v*_max_/*v*_*c*_ for different values of the initial static shear force, 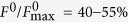 (blue circles), 70–85% (black diamonds) and 85–100% (red squares). *Insert:* Slope *α* of Δ*x*/Δ*x*_0_ vs. log(*v*_max_/*v*_*c*_). The black line is a guide for the eye. [ceramic beads, *d* = 425–600 *μ*m, *ρ* = 3.85 × 10^3^ kg m^−3^, *k* = 870 N/m, *h* = 15 mm, *R*_*H*_ = 42 ± 8%].

Now that (*Aω*) has been identified as the parameter controlling the transition between the stick-slip motion and continuous sliding, one can wonder if the velocity is indeed the parameter controlling the triggering of slip events. To answer the question we performed the additional experiment consisting in measuring the slip distance, Δ*x*, resulting from application of a single tap to a static mechanical situation. To do so, the initial condition is prepared by driving the system in the stick-slip regime in absence of vibration and by suddenly stopping the motor. After a short transient, the slider stops and remains at rest, sustaining a static shear-force *F*^0^. Then, a more or less intense and rapid tap is applied to the system in the transverse direction. The mechanical disturbance is characterized by recording the signal from the accelerometers embedded in the granular layer. Figure 4 displays Δ*x*, normalized by the slip distance 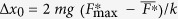 without vibration, for different normalized values 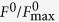 of the initial static shear force, where 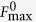 here denotes the maximum force sustained by the slider in the stick-slip regime, without vibration. In accordance with the hypothesis of events triggered by the velocity, we observe, for a given value of 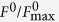, that Δ*x*/Δ*x*_0_ is a function of *v*_max_, the maximum of the velocity associated with the disturbance, and not of the acceleration. We observe a linear dependence on log (*v*_max_/*v*_*c*_), where *v*_*c*_ takes the same value than the threshold velocity reported in the previous experimental configuration, *v*_*c*_ ≃ 100 *μ*m s^−1^. The slope increases with the initial static load, 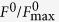 (Fig. 4, inset).

## Discussion and Conclusion

We report strong evidences of the vibration velocity as the parameter governing the response of granular systems subjected to mechanical disturbances. The small value of the critical velocity (*v*_*c*_ ≃ 100 *μ*m s^−1^) which marks the transition between stick-slip motion and continuous sliding is puzzling. We did not observe any significant dependence on the normal load (slider weight) in the experimental range. We thus propose to compare the energy provided by the vibrations to the potential energy barrier a grain must overcome to provoke the transition from the jammed, stick phase to the unjammed, continuous slip. By noting *ξ* the height over which the grains have to jump, we can write the following scaling: 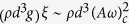, where (*Aω*)_*c*_ = *v*_*c*_ is the critical velocity provided by the vibrations at which the system is unjammed. We thus find *ξ*_*c*_ ~ (*Aω*)_*c*_/*g* ≃ 10^−9^ m, of the order of the typical size of an asperity at the grain surface. The vibrations therefore play the role of a temperature, providing enough thermal agitation to overcome the energy barrier which jams the system.

The critical velocity does not seem to depend significantly on any of the parameters explored in our system. Additional experiments with horizontal vibrations in the sliding direction lead to a velocity threshold of about 60 *μ*m s^−1^. Although different from the threshold reported in the case of horizontal vibrations transversal to the slider motion, both values are of the same order of magnitude. Due to experimental constraints, it is not possible to impose vertical vibrations to our system. However, following the previous scaling arguments, we expect a similar conclusion. These results are in agreement with the very low vibration threshold for experimental unjamming of a granular hopper^16^, where the authors report a critical normalized acceleration of 0.06, at a frequency of 350 Hz, leading to a corresponding velocity of 250 *μ*m s^−1^, of the same order than *v*_*c*_.

The qualitative interpretation proposed with the above scaling does not take into account the normal load, which is small in our experiment (of the order of 50 Pa) and thus does not play a significant role. Interestingly, if a normal force is applied, it should be taken into account instead of the sole gravity in the above energy balance. This is likely to increase the velocity threshold, and could explain field observations for earthquakes, which report an increase of the dynamic triggering threshold velocity with the fault load^18^. Note that in our experiments, we observe a monotonous decrease in both the maximum and average force sustained by the slider (see Fig. 3). Whichever the velocity of the mechanical disturbances, the frictional properties of the granular bed are altered. To the contrary, experiments with large loads (0.1–10 MPa) point out a decrease of the maximum and average force only when the intensity of the mechanical disturbances is large enough^4^. The apparent discrepancy with these experiments, which aim at modelling fault gouge, is likely due to the large difference in the normal load.

The specific influence of vibrations on a sheared granular medium can be seen in the more general frame of jamming and yielding of granular media^8^,^24^,^25^. Dense granular beds are athermal, out-of-equilibrium systems. Although it has been shown that such systems exhibiting jamming are “activated”, in a way similar to thermal systems^7^, it is still debated whether the concept of an effective temperature, specific to equilibrium statistical mechanics, can apply^26^. Here, we show that a granular system under shear may experience a transition from a periodically jammed state (stick-slip) to a large sliding event, triggered by a small vibration. The vibration velocity is the controlling parameter, and its threshold for continuous sliding corresponds to the energy necessary for a grain to jump over a bead asperity. This result underlines, once again, the crucial importance of small, local rearrangements (here, of the order of a few nanometers) on the macroscopic behaviour of the system.

## Additional Information

**How to cite this article**: Lastakowski, H. *et al.* Granular friction: Triggering large events with small vibrations. *Sci. Rep.* **5**, 13455; doi: 10.1038/srep13455 (2015).
